# Self-Compassion, Perceived Stress, and Trauma-Related Symptoms in Adolescents Exposed to an Earthquake

**DOI:** 10.3390/children13070873

**Published:** 2026-06-30

**Authors:** Elif Abanoz, Beyza Karatas Bozok, Ayla Uzun Cicek, Baran Calisgan, Mehmet Karadag

**Affiliations:** 1 Department of Child and Adolescent Psychiatry, Faculty of Medicine, Trabzon University, 61335 Trabzon, Turkey; 2 Department of Child and Adolescent Psychiatry, Sivas State Hospital, 58040 Sivas, Turkey; 3 Department of Child and Adolescent Psychiatry, Faculty of Medicine, Cumhuriyet University, 58140 Sivas, Turkey; 4 Department of Child and Adolescent Psychiatry, Faculty of Medicine, Gaziantep University, 27410 Gaziantep, Turkey

**Keywords:** self-compassion, perceived stress, trauma, earthquake, adolescents

## Abstract

**Highlights:**

**What are the main findings?**

- Adolescents exposed to earthquake-related traumatic experiences reported higher perceived stress and trauma-related symptoms and lower self-compassion.
- Self-compassion was negatively associated with perceived stress and trauma-related symptoms, while perceived stress was positively associated with trauma reactions.

**What are the implications of the main findings?**

- The findings highlight the relevance of self-compassion in understanding adolescents’ psychological responses following natural disasters.
- Self-compassion-focused psychosocial approaches may be considered in post-disaster support programs for adolescents exposed to severe traumatic experiences.

**Abstract:**

**Background:** Adolescents exposed to large-scale natural disasters are at increased risk for psychological distress, yet individual psychological characteristics associated with stress and trauma responses remain insufficiently explored. Self-compassion has been suggested as a relevant factor in stress regulation and emotional adjustment. This study aimed to examine the associations between self-compassion, perceived stress, and trauma-related reactions in adolescents exposed to an earthquake. **Methods:** The sample consisted of 3362 adolescents (57.3% female) aged 14–17 years (mean age = 15.01 ± 0.97 years) who were exposed to the 2020 Elazığ–Sivrice earthquake. Participants completed the Perceived Stress Scale (PSS), the Self-Compassion Scale–Short Form (SCS-SF), and the Child Post-Traumatic Stress Reaction Index (CPTS-RI). Group comparisons and correlation analyses were conducted to examine relationships among the study variables and earthquake-related characteristics. **Results:** Adolescents residing in severely and moderately damaged areas reported significantly higher perceived stress and lower self-compassion compared to those in mildly damaged areas. Self-compassion was negatively correlated with trauma-related symptoms, whereas perceived stress showed a positive association with trauma reactions. Female adolescents reported higher perceived stress and trauma-related symptoms and lower self-compassion than males. **Conclusions:** The findings underscore the relevance of self-compassion in understanding adolescents’ psychological responses to earthquake-related stress and trauma and suggest that self-compassion may be considered in post-disaster psychological support efforts.

## 1. Introduction

Individuals may be exposed to a wide range of potentially traumatic experiences throughout life, including interpersonal violence, serious accidents, life-threatening illnesses, bereavement, war-related events, and natural disasters. Exposure to such experiences may result in substantial psychological distress and trauma-related symptoms, although individual responses vary considerably [1]. Among these potentially traumatic experiences, natural disasters represent a major public health concern because they often affect large populations simultaneously and disrupt social, economic, and environmental systems [2].

Natural disasters, particularly earthquakes, have profound effects on societies, resulting in substantial physical destruction as well as widespread psychological consequences. Earthquakes occur suddenly and unpredictably, often exposing individuals to highly stressful and potentially traumatic experiences. Such events have been associated with a range of adverse psychological outcomes, including post-traumatic stress disorder (PTSD), anxiety, depression, elevated levels of perceived stress, and diminished quality of life. However, individuals’ psychological responses to disaster-related trauma vary considerably, depending on factors such as the degree of exposure, available resources, and individual coping characteristics [3,4,5].

Perceived stress refers to the degree to which individuals perceive situations in their lives as unpredictable, uncontrollable, and overwhelming, and reflects individuals’ appraisal of available coping resources in response to environmental demands [6,7]. Following sudden and large-scale events such as earthquakes, perceived stress levels may differ substantially across individuals, even among those exposed to similar objective conditions [4]. In this context, growing attention has been directed toward individual psychological characteristics that are associated with stress responses and emotional adjustment. One such characteristic is self-compassion, which refers to an individual’s tendency to approach personal suffering, perceived inadequacies, and adverse experiences with kindness, understanding, and emotional balance [8].

Previous research has consistently demonstrated that higher levels of self-compassion are associated with lower levels of perceived stress, anxiety, and psychological distress across diverse populations [9,10]. Across multiple studies, self-compassion has been linked to more adaptive emotional responses and greater psychological resilience under stress [11,12]. These associations suggest that self-compassion may represent an important psychological characteristic related to how individuals experience and report stress following challenging life events. Nevertheless, most of the existing literature has focused on general stressors or interpersonal and self-relevant adversities, while fewer studies have examined self-compassion in the context of large-scale, externally driven traumatic events such as earthquakes.

Adolescence is characterized by ongoing neurobiological, cognitive, emotional, and social development. Heightened emotional reactivity, evolving coping capacities, and increased sensitivity to environmental stressors may increase vulnerability to the psychological consequences of traumatic experiences [13]. Previous studies have reported elevated rates of posttraumatic stress symptoms, anxiety, depression, and functional impairment among adolescents following natural disasters [2,13]. In addition, earthquake-related experiences such as severe property damage, displacement, injury, loss of loved ones, and disruption of daily routines have been associated with greater psychological distress and trauma-related symptoms in young people [2,13]. Previous research has also reported gender differences in post-disaster psychological outcomes, with female adolescents generally reporting higher levels of perceived stress and trauma-related symptoms than males [13].

Despite increasing interest in self-compassion as a factor associated with stress and well-being and growing research on psychological outcomes following earthquakes [14,15], research specifically examining the relationships among perceived stress, self-compassion, and trauma-related symptoms in adolescent earthquake survivors remains limited. To our knowledge, the relationships among perceived stress, self-compassion, and trauma-related symptoms in the context of earthquake-related experiences, damage severity, and gender differences have not been adequately investigated among adolescents exposed to a major earthquake.

The present study aimed to examine the relationships between perceived stress, self-compassion, and trauma-related symptoms among adolescents exposed to the 2020 Elazığ–Sivrice earthquake. In addition, differences in perceived stress, self-compassion, and trauma-related symptoms according to earthquake-related experiences, damage severity, and gender were investigated. Given previous findings suggesting associations between self-compassion, stress, and psychological adjustment, the study also sought to explore the extent to which self-compassion and perceived stress were associated with trauma-related symptom severity in this population. It was hypothesized that higher perceived stress would be associated with greater trauma-related symptom severity, whereas higher self-compassion would be associated with lower levels of perceived stress and trauma-related symptoms. It was further hypothesized that adolescents reporting more severe earthquake-related experiences and damage would exhibit higher perceived stress and trauma-related symptom levels and lower self-compassion scores.

## 2. Materials and Methods

### 2.1. Participants and Procedure

This study included 3362 adolescents aged 14–17 years who reported being exposed to the 6.8 Mw Elazığ–Sivrice earthquake in Türkiye in 2020. Data collection was conducted approximately two years after the earthquake. Eligible participants were adolescents attending the selected high schools who were able to adequately comprehend and complete the self-report questionnaires. Adolescents with a self-reported current or past psychiatric diagnosis or history of psychiatric treatment were excluded from the study. Questionnaires containing missing responses on one or more study variables (*n* = 147) were excluded during data screening prior to data entry and statistical analyses.

Participants were selected from nine high schools in the province using a school-based sampling approach. Information regarding earthquake-related damage severity was obtained from official post-earthquake damage assessments provided through the local governorate, and neighborhoods in the city were classified as having mild, moderate, or severe levels of earthquake-related damage. Based on this classification, high schools located within these neighborhoods were identified, and three schools were selected from each damage category. During school selection, schools were chosen to achieve reasonable comparability with respect to the general sociodemographic characteristics of the student populations. Before the study was conducted, approval was obtained from the provincial directorate of national education. In the participating schools, the first author contacted school administrators, provided detailed information about the study, and administered the questionnaire forms to adolescents who volunteered to participate in classroom settings. Participants were informed that participation was voluntary and that they could discontinue the questionnaires at any time without consequence.

Following a comprehensive explanation of the study protocol, written informed assent was obtained from all participants, and written informed consent was obtained from their parents or legal guardians. The study was conducted in accordance with the Declaration of Helsinki and Good Clinical Practice for Biomedical Research and was approved by the local ethics committee (Date: 1 September 2022, No: 2022/10-33).

### 2.2. Measures

#### 2.2.1. Demographic Information Form

The Demographic Information Form was developed by the researchers to assess participants’ sociodemographic characteristics (e.g., age, sex, place of residence, and family-related variables) as well as earthquake-related experiences. The form included items addressing exposure-related characteristics and contextual factors associated with the earthquake, and was reviewed by the research team for clarity and content relevance prior to data collection. The Demographic Information Form is available in the Supplementary Materials (Supplementary File S1).

#### 2.2.2. Child Post Traumatic Stress Reaction Index (CPTS-RI)

The Child Post-Traumatic Stress Reaction Index (CPTS-RI) is a 20-item self-report measure designed to assess the severity of posttraumatic stress symptoms in children and adolescents following exposure to traumatic events. Each consists of a five-point Likert-type scale ranging from ‘never’ (0) to ‘a lot of the time’ (4). Total scores range from 0 to 80, with higher scores indicating greater symptom severity. Symptom severity is categorized as mild (12–24), moderate (25–39), severe (40–59), and very severe (≥60) posttraumatic stress reactions. The Turkish adaptation of the scale has demonstrated acceptable validity and reliability in child and adolescent samples and was conducted by Erden et al. [16,17]. The CPTS-RI has been widely used to assess posttraumatic stress symptoms in children and adolescents following exposure to traumatic events and natural disasters [14]. In the current study, the CPTS-RI demonstrated excellent internal consistency (Cronbach’s α = 0.924). Given the focus of the study, the CPTS-RI was administered in the context of participants’ experiences related to the 2020 Elazığ–Sivrice earthquake and was used to assess earthquake-related trauma symptoms.

#### 2.2.3. Self-Compassion Scale–Short Form (SCS-SF)

The Self-Compassion Scale–Short Form (SCS-SF) is a self-report tool with 11 items to assess a person’s self-compassion. Each item is Likert-type, rated from 1 (never) to 5 (always). Items 1, 4, 8, 9, 10 and 11 of the scale are reverse coded. The highest score obtained from the scale is 55. High scores obtained from the scale indicate that the person has a high level of self-compassion. The adaptation of the scale to Turkish, validity and reliability study was conducted by Sarı and Yıldırım [18,19,20]. The SCS-SF has been widely used to assess self-compassion in trauma-exposed populations and studies examining earthquake-related psychological outcomes [15]. In the current study, the SCS-SF demonstrated good internal consistency (Cronbach’s α = 0.791).

#### 2.2.4. Perceived Stress Scale (PSS)

The Perceived Stress Scale (PSS), which was developed to measure the level of situations that the individual accepts as stressful in his/her life, consists of 14 items. Items are rated on a five-point Likert scale ranging from 0 (*never*) to 4 (*very often*), with seven items reverse-coded. The adaptation of the scale to Turkish was done by Eskin et al. [7,21]. The PSS has been used in studies examining perceived stress following earthquake exposure and disaster-related experiences [22]. In the current study, the PSS demonstrated good internal consistency (Cronbach’s α = 0.788).

### 2.3. Statistical Analyses

SPSS software (IBM SPSS, Version 22.0, IBM Corporation, Armonk, NY, USA) was used for the statistical analyses of the data. This version was the licensed statistical software available at the institution during the study period. Normality was evaluated using the Kolmogorov–Smirnov test, visual inspection of histograms and Q–Q plots, and examination of skewness and kurtosis values. The assumptions for parametric analyses were considered acceptable. Homogeneity of variances was assessed using Levene’s test where appropriate. The numerical and categorical data were given as mean ± standard deviation (SD), number (*n*), and percentage (%) as appropriate. Statistical comparisons were conducted with independent-samples *t*-tests, one-way ANOVA, or χ^2^ tests (chi-square tests). For analyses involving more than two groups, post hoc multiple-comparison procedures were performed following significant overall group differences. Tukey HSD and Bonferroni procedures were applied where appropriate to identify pairwise group differences while controlling for multiple comparisons. The statistical methods applied in the present study have also been used in recent research investigating psychological responses following earthquakes [14,15]. Correlations were evaluated using Pearson correlation analysis. Scatterplots were visually inspected prior to Pearson correlation analyses to assess the presence of influential outliers. No influential outliers were identified. A *p*-value of <0.05 was considered statistically significant in all analyses.

## 3. Results

### 3.1. Socio-Demographic and Familial Characteristics of Participants

The study included 1928 females (57.3%) and 1434 males (42.7%), with a mean age of 15.01 ± 0.97 years. The sociodemographic characteristics of the participants are presented in Table 1.

### 3.2. Traumatic Experiences of Participants in the Earthquake

Participants’ traumatic experiences related to the earthquake were assessed based on self-report. Following the earthquake, 1366 participants (40.6%) reported having to evacuate their homes. Additionally, 23 participants (0.7%) reported that a family member had died or gone missing, and 81 participants (2.4%) reported that a family member had been injured. Furthermore, 6.0% of participants (*n* = 202) reported marked damage to their homes, and 1.3% (*n* = 43) reported marked property loss.

Regarding exposure to distressing events during the earthquake, 803 participants (23.9%) reported directly watching such events, 660 participants (19.6%) reported hearing about these events, and 1899 participants (56.5%) reported both watching and hearing about distressing events. These findings are presented in Table 2.

### 3.3. Relationships Between PSS, SCS-SF, and CPTS-RI Scores

Pearson correlation analysis revealed that SCS-SF scores were significantly and negatively correlated with both CPTS-RI and PSS scores (r = −0.477, *p* < 0.001; r = −0.525, *p* < 0.001, respectively). Additionally, CPTS-RI scores were positively correlated with PSS scores (r = 0.472, *p* < 0.001). The correlation results are presented in Table 3.

### 3.4. Comparison of Scale Scores Used According to Damage Status

Participants were categorized into three regional groups based on earthquake-related damage severity: severe damage (*n* = 1175), moderate damage (*n* = 1145), and mild damage (*n* = 1042). The groups were generally comparable with respect to the demographic and earthquake-related exposure characteristics evaluated in the study, with no statistically significant differences observed across groups (all *p* > 0.05).

However, significant differences were observed in scale scores. Post hoc Tukey analyses indicated that PSS scores were significantly higher in the severe and moderate damage groups compared to the mild damage group (*p* = 0.018). Similarly, SCS-SF scores were significantly lower in the severe and moderate damage groups than in the mild damage group (*p* < 0.001). No significant differences were observed in CPTS-RI scores across damage severity groups (*p* > 0.05). These results are shown in Table 4.

### 3.5. Comparison of Scale Scores by Gender

Sociodemographic characteristics and earthquake-related traumatic experiences did not differ significantly by gender (all *p* > 0.05). However, females demonstrated significantly higher CPTS-RI and PSS scores and significantly lower SCS-SF scores compared to males (all *p* < 0.001). Detailed results are presented in Table 5.

### 3.6. Comparison of Scale Scores According to Traumatic Experiences in the Earthquake

Comparisons of CPTS-RI, PSS, and SCS-SF scores according to earthquake-related experiences are presented in Table 6. Participants who were required to evacuate their homes reported significantly higher CPTS-RI (*p* < 0.001) and PSS scores (*p* = 0.008) than those who did not, whereas SCS-SF scores did not differ significantly (*p* = 0.083). Participants reporting the death or disappearance of a family member had higher CPTS-RI scores (*p* = 0.032), with no significant differences in PSS or SCS-SF scores (*p* > 0.05). Similarly, participants reporting marked housing damage or property loss showed significantly higher CPTS-RI and PSS scores (all *p* < 0.05), while SCS-SF scores did not differ significantly. Regarding exposure to distressing events, participants who were exposed to both visual and auditory earthquake-related distressing experiences exhibited significantly higher CPTS-RI scores than those who only watched or only heard about such events (*p* < 0.001).

## 4. Discussion

The present study identified several notable patterns in adolescents exposed to an earthquake. Higher levels of perceived stress and lower levels of self-compassion were observed particularly among adolescents residing in severely and moderately damaged areas and among those reporting traumatic experiences. In addition, trauma-related symptoms were negatively associated with self-compassion and positively associated with perceived stress. These findings align with previous research indicating that exposure to traumatic events is linked to heightened stress responses and adverse psychological outcomes in adolescents [23,24].

Perceived stress levels were closely associated with adolescents’ reported traumatic experiences related to the earthquake. This pattern is consistent with previous studies indicating that exposure to traumatic events is linked to variations in psychological responses, including stress and trauma-related symptoms [25,26,27]. In the present study, experiences such as home evacuation, housing damage, and property loss were associated with higher levels of perceived stress and trauma symptoms. These findings align with prior research identifying post-earthquake housing damage as an important correlate of PTSD risk and psychological distress, particularly through its association with repeated exposure to stressors, loss of safety, financial strain, and reduced social support [28,29]. Similarly, studies examining the psychological consequences of earthquake-related home damage have reported associations with poorer mental health outcomes and diminished quality of life over time [2,30]. In addition, material losses, family-related difficulties, and disruptions in social support following earthquakes have been shown to co-occur with increased psychological difficulties in adolescents [13,31]. These findings suggest that earthquake-related physical damage may be associated with adolescents’ psychological experiences and may co-occur with disruptions in living conditions, perceived safety, and social stability.

Significant differences were observed according to the degree and modality of exposure to traumatic experiences. Traumatic reactions and perceived stress levels were higher, while self-compassion levels were lower, among participants who reported both watching and hearing about distressing events, compared to those with more limited exposure. This pattern suggests that multiple forms of exposure are associated with more pronounced psychological responses in adolescents. Consistent with prior research, visual or auditory exposure to traumatic events has been shown to be associated with increased risk of PTSD and other psychological difficulties [27,32]. Moreover, several studies have demonstrated that direct exposure is not a necessary condition for trauma-related symptoms, as witnessing injury or death—particularly in the context of earthquakes—has also been linked to elevated PTSD symptoms [14,17,33,34,35]. The emotional impact of such exposure may be further intensified when the affected individual is a family member or acquaintance, which has been associated with greater psychological burden [29]. In line with this literature, adolescents in the present study who reported the injury, loss, or disappearance of family members exhibited higher levels of trauma-related symptoms. Overall, these findings highlight the relevance of indirect trauma exposure as a factor associated with trauma-related outcomes and emphasize the importance of considering exposure modality when developing psychosocial support strategies for adolescents following natural disasters.

Across damage severity groups, higher perceived stress and lower self-compassion were observed in more severely affected areas. Self-compassion was negatively associated with perceived stress, while trauma-related symptoms showed the opposite pattern. These findings indicate that self-compassion is closely associated with adolescents’ psychological responses to stress and trauma following a major adverse event. Previous research has consistently highlighted self-compassion as an important psychological characteristic related to coping with stress and trauma [9,15,36]. Studies have shown that higher levels of self-compassion are associated with greater emotion regulation, psychological flexibility, and more adaptive coping responses in stressful contexts [37,38]. Similarly, individuals with higher self-compassion tend to report lower levels of psychopathological symptoms, including depression, anxiety, and stress, and to display more balanced emotional responses when facing traumatic experiences [38,39]. Furthermore, a substantial body of literature suggests that self-compassion is associated with trauma-related outcomes and is commonly incorporated into psychotherapeutic approaches addressing PTSD symptoms [38,39,40,41]. Self-compassion has also been associated with emotional regulation processes and psychological resilience following trauma [15,42]. In line with this literature, the present findings highlight the relevance of self-compassion in understanding adolescents’ stress and trauma-related experiences after natural disasters such as earthquakes and suggest that self-compassion may be an important psychological characteristic associated with adolescents’ responses to earthquake-related trauma.

Gender differences emerged as a notable finding in the present study. Female adolescents reported higher levels of trauma-related symptoms and perceived stress, along with lower levels of self-compassion, compared to male adolescents. This pattern is consistent with previous research indicating that females tend to report greater trauma-related distress and higher perceived stress following adverse events [29,43,44]. Such differences have been discussed in the literature in relation to a range of biological, psychological, and social factors, including differential emotional processing, stress responses, and gender-related socialization. Although some studies have suggested that females generally report higher levels of self-compassion [45,46,47], other research has reported mixed findings regarding gender differences in self-compassion, particularly among adolescents [48,49,50,51,52]. The lower self-compassion levels observed among female participants in the present study may be understood in the context of their higher reported trauma reactions and perceived stress, as well as potential methodological and contextual factors influencing self-report across genders. Consistent with prior findings, self-compassion was negatively associated with psychopathological indicators such as stress and trauma-related symptoms, supporting its relevance as a psychological characteristic linked to emotional well-being [38,50,53,54]. Overall, these findings underscore the importance of considering gender differences when interpreting adolescents’ psychological responses to natural disasters.

### Limitations

Several limitations should be acknowledged. First, the absence of a non-exposed control group limits comparative interpretations. Second, the cross-sectional design restricts conclusions to observed associations rather than causal relationships and precludes conclusions regarding the temporal direction of the observed relationships. Third, reliance on self-report measures may have introduced recall and reporting biases. In addition, data were collected approximately two years after the earthquake, which may reflect more enduring psychological responses rather than acute reactions and may have been influenced by experiences occurring during the intervening period. Although adolescents with a self-reported history of psychiatric diagnosis or psychiatric treatment were excluded, this exclusion criterion may have limited the generalizability of the findings, as adolescents with pre-existing psychiatric vulnerabilities may be particularly susceptible to adverse psychological outcomes following natural disasters and may have exhibited different patterns of perceived stress, self-compassion, and trauma-related symptoms. Information regarding post-earthquake psychosocial support services or non-clinical psychological interventions was not collected. Therefore, the potential influence of such interventions on perceived stress, self-compassion, and trauma-related symptoms could not be evaluated. Furthermore, the school-based sampling approach may have limited the representativeness of the sample, as adolescents who were not attending school at the time of data collection—including those who migrated following the earthquake or experienced more severe psychosocial difficulties—may have been underrepresented. Therefore, the findings may not fully capture the experiences of the most severely affected adolescents. Similar methodological limitations have also been reported in previous earthquake-related mental health studies [15,27].

## 5. Conclusions

The present study examined the relationships between perceived stress, self-compassion, and trauma-related symptoms among adolescents exposed to the 2020 Elazığ–Sivrice earthquake. Consistent with the study hypotheses, higher perceived stress was associated with greater trauma-related symptom severity, whereas higher self-compassion was associated with lower levels of perceived stress and trauma-related symptoms. In addition, more severe earthquake-related experiences and damage were associated with higher perceived stress and trauma-related symptom levels. By highlighting these associations, the study contributes to the literature on adolescents’ psychological responses following large-scale natural disasters.

Overall, the results underscore the relevance of self-compassion in understanding adolescents’ stress and trauma-related experiences following natural disasters. While self-compassion was closely associated with perceived stress and trauma-related symptoms, further longitudinal and intervention-based studies are needed to clarify how these relationships may evolve over time. Future research incorporating multiple informants and diverse study designs may help to deepen understanding of these relationships. The findings also highlight the potential relevance of perceived stress and self-compassion in understanding individual differences in trauma-related psychological responses following natural disasters. In addition, studies examining self-compassion-focused interventions may further clarify their potential relevance in post-disaster psychosocial support programs for adolescents exposed to severe traumatic experiences.

## Figures and Tables

**Table 1:** Socio-demographic and familial characteristics of participants. Data were given as mean ± standard deviation or number (percent%).

Variables	*n* (%) or Mean ± SD
Age (mean-years ± SD)	15.01 ± 0.97
Sex	
Male	1434 (42.7)
Female	1928 (57.3)
Age of mother (mean-years ± SD)	41.30 ± 5.25
Level of education of the mother	
High school and lower	3047 (90.6)
University	315 (9.4)
Maternal psychiatric disorder	130 (3.9)
Age of father (mean-years ± SD)	46.08 ± 5.69
Level of education of the father	
High school and lower	2644 (78.6)
University	718 (21.4)
Paternal psychiatric disorder	73 (2.2)
Family income level	
The minimum wage/less than minimum wage	2278 (67.8)
Above the minimum wage	1084 (32.2)
Family type	
Nuclear	2906 (86.4)
Single-parent	456 (13.6)

Note. Data are presented as mean ± standard deviation (SD) or *n* (%).

**Table 2:** Traumatic experiences of participants in the earthquake. Data were given as mean ± standard deviation or number (percent%).

Variables	*n* (%) or Mean ± SD
Having to leave home after the earthquake	
Yes	1366 (40.6)
No	1996 (59.4)
Family member affected in the earthquake	
Death/missing	23 (0.7)
Injury	81 (2.4)
None of the above	3258 (96.9)
Damage status of house in the earthquake	
Total	202 (6.0)
Severe	116 (3.5)
Moderate	450 (13.4)
Mild	1134 (33.7)
None of the above	1460 (43.4)
Property loss in the earthquake	
Total	43 (1.3)
Severe/Moderate	190 (5.7)
Mild	299 (8.9)
None of the above	2830 (84.2)
Witnessing dramatic events in the earthquake	
Watching about events	803 (23.9)
Hearing about events	660 (19.6)
All of the above	1899 (56.5)

Note. Data are presented as mean ± standard deviation (SD) or *n* (%).

**Table 3:** Relationships between Perceived Stress Scale, Self-Compassion Scale, and Child Post Traumatic Stress Reaction Index scores.

Parameters	CPTS-RI		PSS		SCS-SF	
	**r**	***p***	**r**	***p***	**r**	***p***
CPTS-RI	-	-	0.472	**<0.001**	−0.477	**<0.001**
PSS	0.472	**<0.001**	-	-	−0.525	**<0.001**
SCS-SF	−0.477	**<0.001**	−0.525	**<0.001**	-	-

Note. Pearson correlation analysis was performed. Bold values indicate statistically significant correlations (*p* < 0.05). CPTS-RI = Child Post-Traumatic Stress Reaction Index; PSS = Perceived Stress Scale; SCS-SF = Self-Compassion Scale–Short Form.

**Table 4:** Comparison of scale scores used according to damage status. Data were given as mean ± standard deviation.

	Mild Damage Region (*n* = 1042)	Moderate Damage Region (*n* = 1145)	Severe Damage Region (*n* = 1175)	*p*-Value
CPTS-RI Total Score	25.32 ± 17.82	26.94 ± 18.09	26.52 ± 18.30	0.089
PSS Total Score	26.99 ± 10.09	27.83 ± 10.13	27.99 ± 10.53	**0.018**
SCS-SF Total Score	33.03 ± 9.08	31.75 ± 8.36	31.21 ± 9.67	**<0.001**

Note. Data are presented as mean ± standard deviation. Group differences were tested using one-way ANOVA. Bold values indicate statistically significant differences (*p* < 0.05).

**Table 5:** Comparison of scale scores by gender. Data were given as mean ± standard deviation.

	Male (*n* = 1434)	Female (*n* = 1928)	*p*-Value
CPTS-RI Total Score	20.37 ± 15.54	30.69 ± 18.59	**<0.001**
PSS Total Score	25.30 ± 9.80	29.36 ± 10.27	**<0.001**
SCS-SF Total Score	33.89 ± 8.52	30.53 ± 9.23	**<0.001**

Note. Data are presented as mean ± standard deviation. Group differences were tested using independent-samples *t*-test. Bold values indicate statistically significant differences (*p* < 0.05). SD = standard deviation.

**Table 6:** Comparison of scale scores according to traumatic experiences in the earthquake. Data were given as mean ± standard deviation.

	CPTS-RI Total Score	PSS Total Score	SCS-SF Total Score
Having to leave home in the earthquake			
Yes	28.95 ± 18.78	28.11 ± 10.48	31.67 ± 9.06
No	24.47 ± 17.37	27.30 ± 10.10	32.16 ± 9.10
*p*-value	**<0.001**	**0.008**	0.083
Family members affected in the earthquake			
Death/missing	32.35 ± 18.43	25.65 ± 11.03	30.48 ± 7.92
Injury	30.33 ± 18.95	28.25 ± 10.72	30.90 ± 8.90
None of the above	26.15 ± 18.05	27.62 ± 10.25	32.00 ± 9.10
*p*-value	**0.032**	0.673	0.563
Damage status of house in the earthquake			
Total	31.63 ± 18.84	28.29 ± 9.91	31.63 ± 8.98
Severe	29.73 ± 19.22	28.26 ± 10.53	31.73 ± 8.73
Moderate	28.65 ± 20.88	28.09 ± 10.12	31.43 ± 8.89
Mild	27.23 ± 17.49	26.95 ± 10.47	32.37 ± 9.82
None of the above	23.58 ± 17.43	26.12 ± 9.93	32.42 ± 9.24
*p*-value	**<0.001**	**0.025**	0.075
Property loss in the earthquake			
Total	32.64 ± 19.15	30.33 ± 9.38	30.88 ± 11.56
Severe/Moderate	31.77 ± 19.13	29.27 ± 11.16	31.66 ± 8.48
Mild	29.04 ± 18.53	27.29 ± 9.38	31.27 ± 9.37
None of the above	25.38 ± 17.81	27.52 ± 10.16	32.06 ± 9.09
*p*-value	**<0.001**	**0.009**	0.506
Witnessing dramatic events in the earthquake			
Watching about events	29.75 ± 18.36	29.73 ± 10.17	30.54 ± 8.83
Hearing about events	21.36 ± 15.73	25.93 ± 9.91	33.49 ± 8.75
All of the above	35.10 ± 19.07	29.91 ± 10.42	29.53 ± 9.35
*p*-value	**<0.001**	**<0.001**	**<0.001**

Note. Data are presented as mean ± standard deviation. Group differences were tested using one-way ANOVA. Bold values indicate statistically significant differences (*p* < 0.05).

## Data Availability

The data that support the findings of this study are available on request from the corresponding author. The data are not publicly available due to privacy or ethical restrictions.

## Supplementary Materials

The following supporting information can be downloaded at: https://www.mdpi.com/article/10.3390/children13070873/s1, Supplementary File S1: Demographic Information Form.
