# Supplementary material for: Self-Compassion, Perceived Stress, and Trauma-Related Symptoms in Adolescents Exposed to an Earthquake
Source: Children (Basel). 2026 Jun 30;13(7):873. doi: 10.3390/children13070873 (PMC13406952; doi:10.3390/children13070873)
Supplement: Supplementary file 1 [file children-13-00873-s001.zip › children-4348943-supplementary.pdf]

Supplementary File S1. Demographic Information Form

1. Age: _____ years

2. Sex
□ Male
□ Female

3. Mother's age: _____ years

4. Mother's educational level
□ Primary school
□ Middle school
□ High school
□ University

5. Has your mother ever been diagnosed with or received treatment for a psychiatric disorder?
□ Yes
□ No

6. Father's age: _____ years

7. Father's educational level
□ Primary school
□ Middle school
□ High school
□ University

8. Has your father ever been diagnosed with or received treatment for a psychiatric disorder?
□ Yes
□ No

9. Family income level
□ Minimum wage or below
□ Above minimum wage

10. Family structure
□ Nuclear family
□ Single-parent family

11. Did you have to leave your home after the earthquake?
□ Yes
□ No

12. Did any family member die, go missing, or become injured during the earthquake?
□ Death/Missing
□ Injury
□ None of the above

13. What was the level of damage to your home following the earthquake?
□ Total damage
□ Severe damage
□ Moderate damage
□ Mild damage
□ No damage

14. Did your family experience property loss due to the earthquake?
□ Total loss
□ Severe/Moderate loss
□ Mild loss
□ No loss

15. Which of the following best describes your exposure to dramatic events during the earthquake?
□ Witnessed events directly
□ Heard about events from others
□ Both witnessed and heard about events
